# Posttranscriptional regulation of AKT by circular RNA angiomotin- like 1 mediates chemoresistance against paclitaxel in breast cancer cells

**DOI:** 10.18632/aging.102535

**Published:** 2019-12-09

**Authors:** Jian Ma, Ling Fang, Qi Yang, Steven Hibberd, William W. Du, Nan Wu, Burton B. Yang

**Affiliations:** 1 Sunnybrook Research Institute, Sunnybrook Health Sciences Centre, Toronto, Canada; 2 Department of Urology, The Affiliated Yantai Yuhuangding Hospital of Qingdao University, Yantai, China; 3 The First Hospital, Jilin University, Jilin, China; 4 Department of Laboratory Medicine and Pathobiology, University of Toronto, Toronto, Canada

**Keywords:** circular RNA, circAMOTL1, chemoresistance, PAX, AKT

## Abstract

Chemoresistance of triple negative breast cancer against paclitaxel (PAX) is one of the major issues for the patients under chemotherapy. However, the mechanism by which the breast cancer cells are resistant to PAX remains unclear. Here, we identified a circular RNA of angiomotin-like 1 (circAMOTL1) as an important player which may be responsible for the adverse resistance against PAX in breast cancer cells. The circAMOTL1 were overexpressed in MDA-MB-231 breast cancer cells via transfection of circAMOTL1 construct. Overexpression of circAMOTL1 caused significant increase of cell viability, reduction of apoptosis, and enhancement of invasion when MDA-MB-231 cells were exposed to PAX compared to those cells with vector control. Moreover, these resistant effects could be blocked by the application of circAMOTL1 siRNA. In these circAMOTL1 overexpressing cells, we found notable increase of both phosphorylated and total AKT protein, which suggested that AKT might be the downstream factor mediating the resistant effects. Consequently, the gene and protein expression of AKT related pro-apoptotic (BAX and BAK) and anti-apoptotic (BCL-2) factors were significantly changed by circAMOTL1 as well. These results suggest circAMOTL1 may play an important role in the PAX resistance of breast cancer cells via regulation of AKT pathway, facilitation of anti-apoptotic protein and inhibition of pro-apoptotic protein. While providing a new mechanism of PAX resistance in breast cancer cells, our findings may lay groundwork for a novel therapeutic target of the breast cancer treatment in the future.

## INTRODUCTION

Breast cancer is one of the most common invasive cancers among women worldwide. Over 1 million cases are diagnosed yearly and approximately 40 thousand women die from breast cancer every year [1]. Among all types of breast cancer, triple negative breast cancer has the worst prognosis due to its refractoriness to hormone therapy. Currently, chemotherapy is the major therapeutic strategy for the patients with triple negative breast cancer after surgery. However, drug resistance to chemotherapy appears to be the major obstacle preventing breast cancer patients from having a better prognosis once exposed to this type of treatment. Paclitaxel (PAX), also known as Taxol, is one of the agents belonging to taxanes family, which is the recommended first-line treatment for metastatic breast cancer [2]. It has been well documented in the literature that PAX acts by stabilizing microtubules and thwarting the cancer cells from division [3]. Although PAX is effective and achieves an impressive initial response, the majority of patients with advanced cancer eventually develop resistance to PAX after 6 to 10 months of treatment [4, 5]. Therefore, understanding the mechanisms by which the breast cancer cells develop resistance to PAX is of great importance for managing the dilemma of treating patients with breast cancer.

Recently, thousands of endogenously expressed circular RNA isoforms have been revealed [6, 7]. Circular RNA is a large group of transcripts that forms a covalently closed continuous loop [8, 9]. Circular RNAs are abundant in diverse species and exert a variety of biological functions [10–14]. A variety of circular RNAs were validated to act as natural sponges to arrest the activity of various microRNA [15–17]. Other than binding with microRNA, our previous studies have demonstrated that circular RNAs could also bind with proteins and thus regulate the cell function [18–21]. For example, we found Foxo3 circular RNA (circFoxo3) could bind to cell division protein kinase 2 (CDK2) and cyclin-dependent kinase inhibitor 1 (p21) to block the cell cycle progression [18], interact with senescence and stress-associated proteins to increase cellular senescence [19], or bind with mdm2 and p53 to induce cell apoptosis [20]. Besides circFoxo3, we also found notable functional activities of another circular RNA, circular angiomotin-like 1 (circAMOTL1) in breast cancer models [22]. CircAMOTL1 played important roles in promoting cell proliferation and survival via interaction with c-myc proteins in breast cancer cells [22]. However, whether circAMOTL1 takes an essential part in chemoresistance against PAX treatment in breast cancer cells was never examined.

AKT kinase activity is significantly boosted in breast cancer, which promotes tumorigenesis and metastasis [23]. Moreover, the constitutive activation of AKT is associated with resistance to multiple chemotherapeutic agents [24]. Several previous studies have suggested that AKT is one of the key mediators for chemoresistance against PAX in cancer cells [25, 26]. Further characterization of the regulating mechanism of AKT-dependent signaling in chemotherapeutic resistance may help to define the precise direction to develop novel agents that have great clinical utility for breast cancer treatment. Therefore, in the present study, we explored whether circAMOTL1 was involved in the chemoresistance against PAX in breast cancer cells and how circAMOTL1 participated in the process by investigating its possible influence on AKT and its signaling pathway.

## RESULTS

### CircAMOTL1 mediated the drug resistance against PAX in breast cancer cells

**Figure 1:**
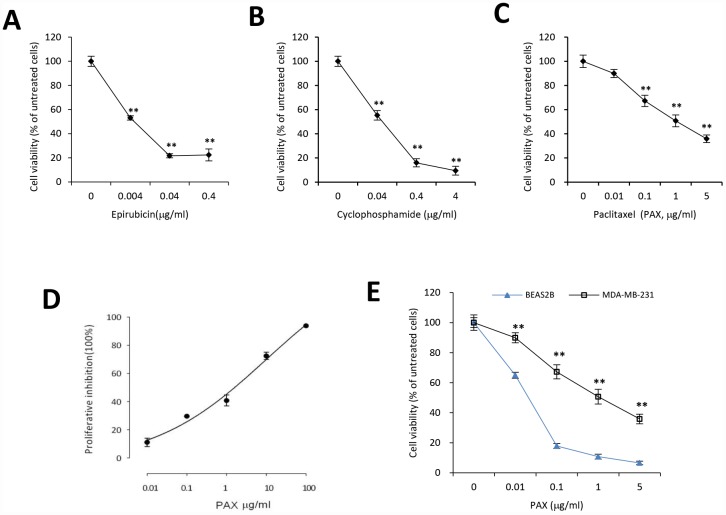
**Chemoresistance of breast cancer MDA-MB-231 cells against PAX, cyclophosphamide or epirubicin.** MDA-MB-231 cells were treated with different concentrations of epirubicin (**A**), cyclophosphamide (**B**), or PAX (**C**) for 24 hours, respectively. Chemoresistance was shown by cell viability. n=3 for each treatment. **p<0.01 compared to untreated cells. (**D**) The half maximal inhibitory concentration (IC50) of PAX in MDA-MB-231 cells was determined by using CCK8. (**E**) BEAS-2B, the immortalized normal cells, and MDA-MB-231 cells were treated with 0.01, 0.1, 1 or 5 μg/ml PAX respectively for 24 hours. n=3 for each treatment. **p<0.01 compared to the value of BEAS-2B cells at the corresponding time point.

**Figure 2:**
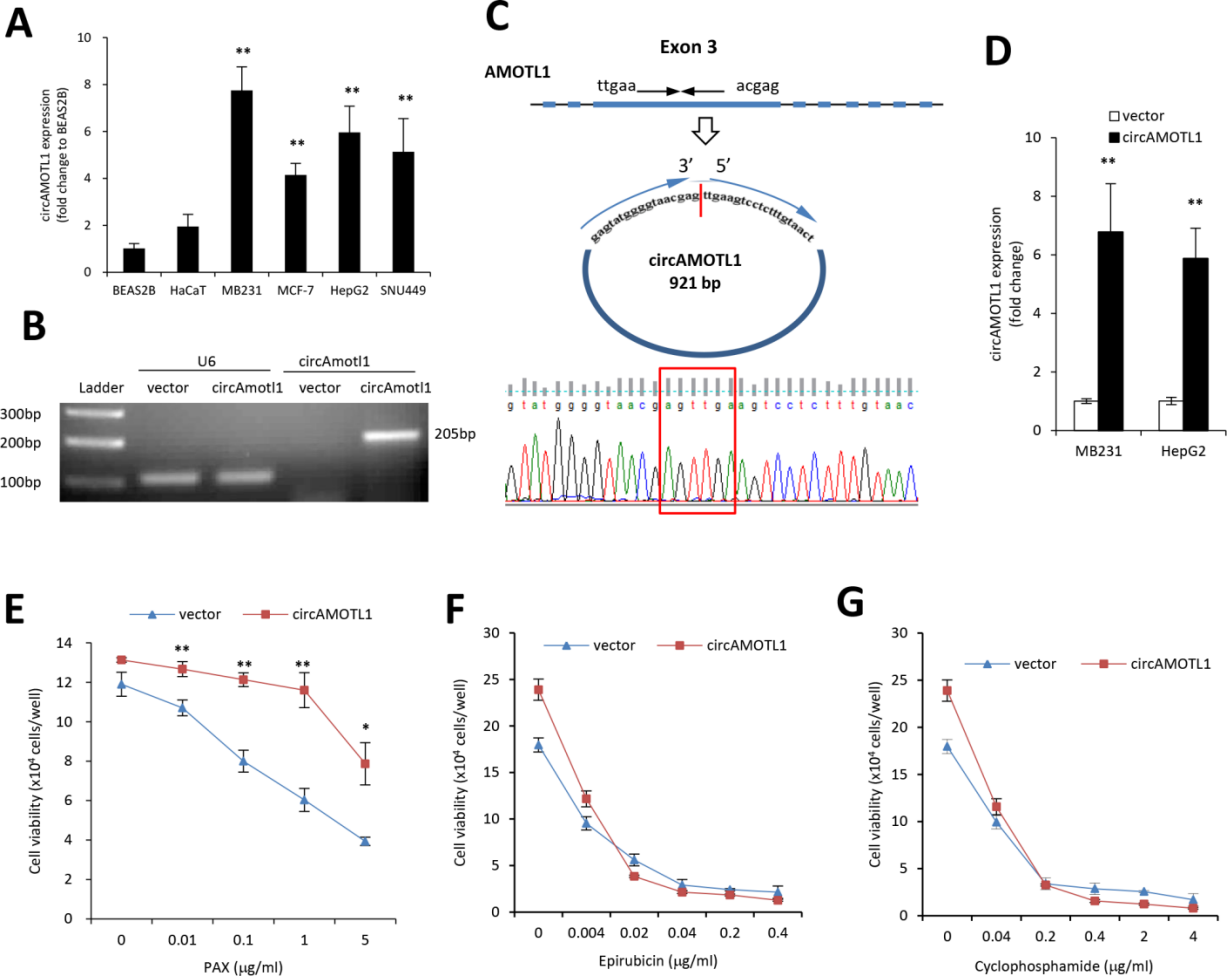
**Role of circular RNA of circAMOTL1 expression in drug resistance.** (**A**) The expression of circAMOTL1 in immortalized normal cells BEAS-2B and HaCaT, and cancer cells MDA-MB-231, MCF-7, HepG2 and SNU449 were examined using real-time PCR (n=4). **p<0.01 compared to BEAS-2B cells. (**B**) The expression of circAMOTL1 was shown in agarose gel after the MDA-MB-231 cells were transfected with circAMOTL1 plasmids or vector control. (**C**) The existence of circAMOTL1 was validated by Sanger sequencing. Red arrow represents “head to tail” junction of circAMOTL1. (**D**) The overexpression of circAMOTL1 was validated by qPCR in the MDA-MB-231 and HepG2 cells. n=6. **p<0.01 compared to vector control. MDA-MB-231 cells transfected with vector or circAMOTL1 plasmids were treated with different concentrations of PAX (**E**), epirubicin (**F**), or cyclophosphamide (**G**) for 24 hours, respectively. Chemoresistance was shown by cell viability. n=3 for each treatment. **p<0.01 compared to untreated cells.

MDA-MB-231 cells are triple negative, claudin-low human breast cancer cells with intermediate response to chemotherapy [27]. We examined the cell viability upon treatment of three different chemotherapeutic drugs, epirubicin, cyclophosphamide, and PAX. Our results indicated that compared to epirubicin (Figure 1A) and cyclophosphamide (Figure 1B), MDA-MB-231 cells were more resistant to PAX (Figure 1C). The IC50 for MDA-MB-231 cells against PAX is 1.219 μg/ml (Figure 1D). In addition, we compared the drug resistance against PAX in an immortalized normal cells BEAS-2B and breast cancer cells MDA-MB-231. The results showed that MDA-MB-231 cells were resistant to PAX while BEAS-2B cells were vulnerable to PAX (Figure 1E). Then, we asked whether there were distinct expressing profiles of circAMOTL1 in these cells. We performed qPCR to measure the expression levels of circAMOTL1 in normal cell lines, such as BEAS-2B and HaCaT and several cancer cell lines, including MDA-MB-231, MCF-7, HepG2 and SNU449 cells. As shown in the Figure 2A, there were significant differences between the expression level of BEAS-2B and other cancer lines.

Next, we used plasmid construct which expressed circAMOTL1 to transfect 293T cells to create the circAMOTL1 overexpressing cells. As expected, our results showed a significantly increased circAMOTL1 expression after transient transfection with circAMOTL1 plasmids compared to vector control (Figure 2B). Subsequently, we confirmed the “head-to-tail splicing” in the RT-PCR product of circAMOTL1 with expected size and identity by using Sanger sequencing (Figure 2C). In addition, we examined the circAMOTL1 levels in MDA-MB-231 cells and HepG2 cells with stable transfection of circAMOTL1 plasmids by using qPCR. Here, we found that the circAMOTL1 expression was remarkably increased in both of these two cell lines (Figure 2D). Next, to confirm whether circAMOTL1 was involved in the above chemoresistance in these breast cancer cells, we measured the cell viability of MDA-MB-231 cells with or without circAMOTL1 overexpression upon the treatment of PAX (Figure 2E), epirubicin (Figure 2F) and cyclophosphamide (Figure 2G). Our results showed that the cells with circAMOTL1 overexpression became much more resistant against PAX but not epirubicin and cyclophosphamide.

### CircAMOTL1 overexpression enhanced the malignant cell behaviors in breast cancer cells exposed to PAX

**Figure 3:**
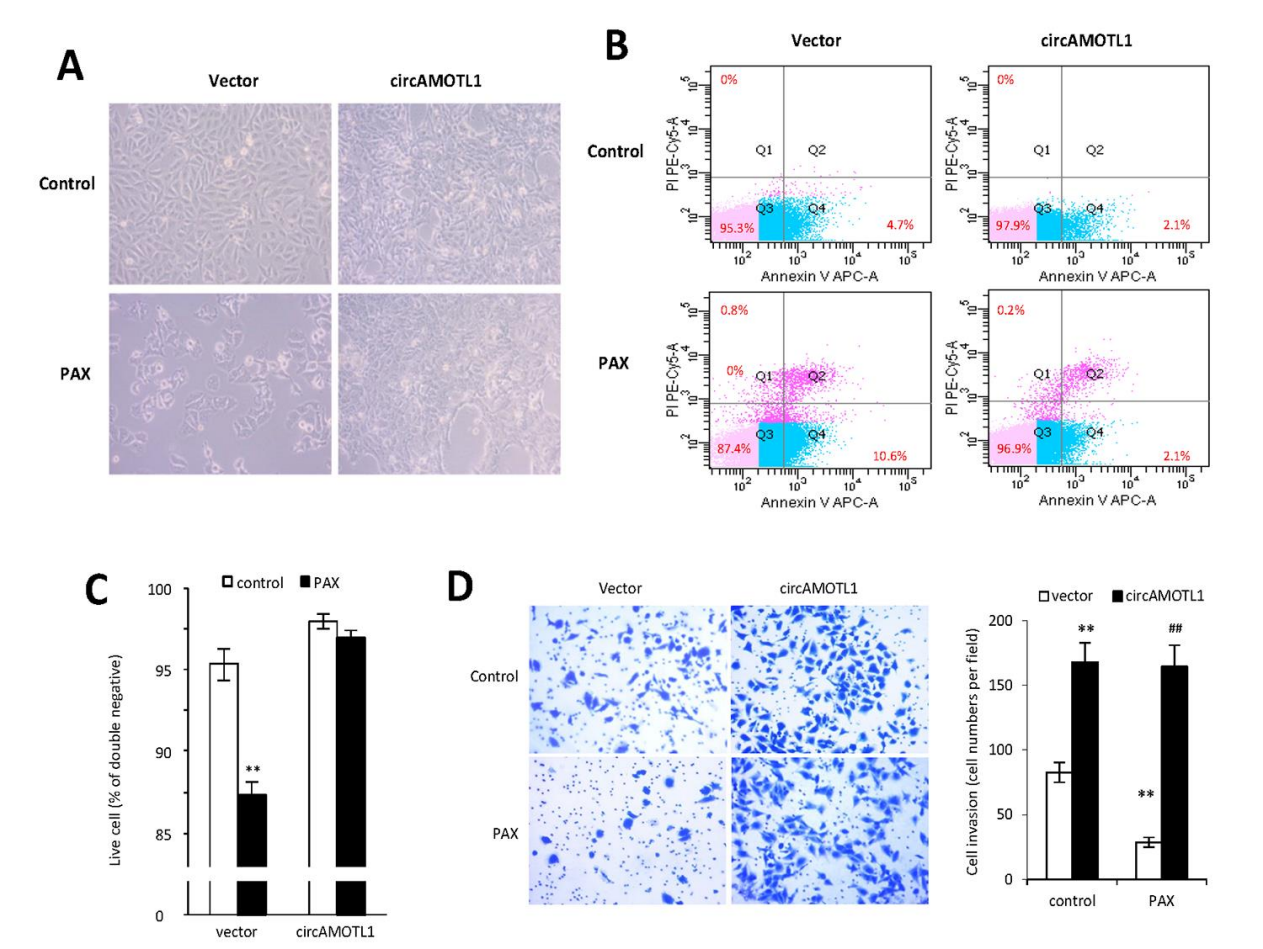
**Effect of circAMOTL1 overexpression on PAX treatment.** MDA-MB-231 cells stably transfected with vector control or circAMOTL1 were treated with 1 μg/ml PAX for 24 hours. (**A**) Cells morphological changes upon PAX treatment. (**B**) Cell apoptosis upon PAX treatment. Cells were stained with Annexin V/PI double staining followed by FACS analysis. Experiments were performed in triplicate. (**C**) Quantitation of double negative cells (alive cells). (**D**) Cell invasive ability upon PAX treatment was measured with Matrigel invasion assay. Experiments were performed in triplicate. **p<0.01 compared to untreated vector control. ^##^p<0.01 compared to PAX treated vector control (n=3).

It was anticipated that PAX treatment would affect cell invasion and apoptosis. To validate the role of circAMOTL1 in PAX resistance, the cell apoptosis and invasion capabilities were examined in MDA-MB-231 cells with stable transfection of circAMOTL1 or vector upon PAX treatment. Our results showed that the cells with circAMOTL1 overexpression exerted enhanced survival ability when exposed to PAX compared to cells with vector control (Figure 3A). In addition, the flow cytometry analysis showed that Annexin V and PI double-stained apoptotic cells were significantly increased in vector control cells after PAX treatment, while the apoptotic cells in circAMOTL1 overexpression group were much less than the vector control group (Figure 3B). As a result, the number of alive cells increased (Figure 3C). Next, we investigated the invasion of these two groups of cells exposed to PAX individually. We found that the invasive ability of circAMOTL1 cells were comparable in treated and untreated groups, while the cell invasion was significantly decreased in vector control cells upon PAX treatment compared to their counterparts without PAX treatment (Figure 3D).

**Figure 4:**
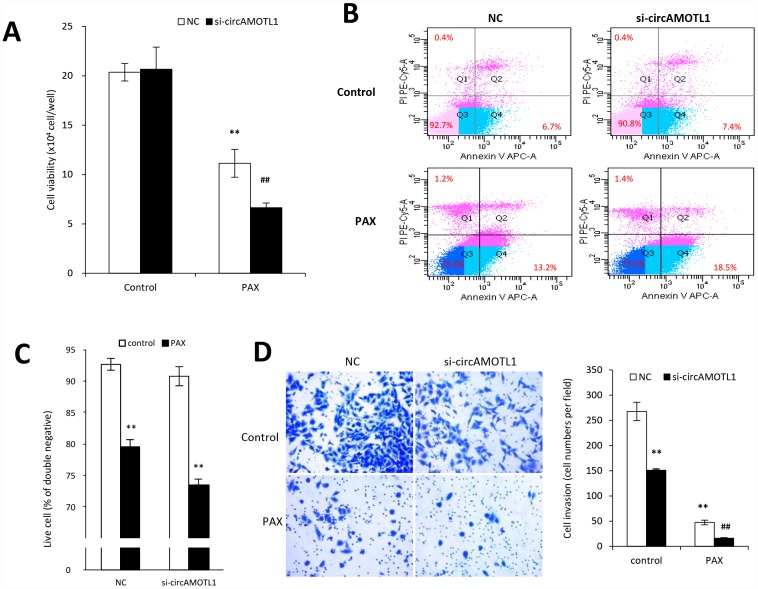
**Effect of circAMOTL1 siRNA on PAX treatment.** MDA-MB-231 cells transfected with negative control or circAMOTL1 siRNA (si-circAMOTL1) were treated with 1 μg/ml PAX for 24 hours. (**A**) Cell viability was detected after siRNA transfection upon PAX treatment. n=5. **p<0.01 compared to untreated negative control. ^##^p<0.01 compared to untreated si-circAMOTL1. (**B**) Cell apoptosis upon PAX treatment was stained with Annexin V/PI double staining followed by FACS analysis. Experiments were performed in triplicate. (**C**) Quantitation of double negative cells (alive cells). (**D**) Cell invasive ability upon PAX treatment was measured with matrigel invasion assay. Experiments were performed in triplicate. **p<0.01 compared to untreated negative control. ^##^p<0.01 compared to untreated si-circAMOTL1.

To further confirm the specific role of circAMOTL1 in PAX-resistance, we transfected the circAMOTL1 siRNA into MDA-MB-231 cells followed by PAX treatment. The results showed that PAX significantly decreased the cell viability if endogenous circAMOTL1 was silenced by siRNA (Figure 4A). Meanwhile, the number of apoptotic cells was significantly increased in the circAMOTL1 siRNA transfected MDA-MB-231 cells exposed to PAX treatment compared to the scramble siRNA cells (Figure 4B), while the number of alive cells decreased (Figure 4C). Moreover, the MDA-MB-231 cells treated with circAMOTL1 siRNA showed less cell invasion and also their invasive ability could be further minimized when PAX was added (Figure 4D).

### Role of AKT pathway in circAMOTL1 mediated PAX-resistance

**Figure 5:**
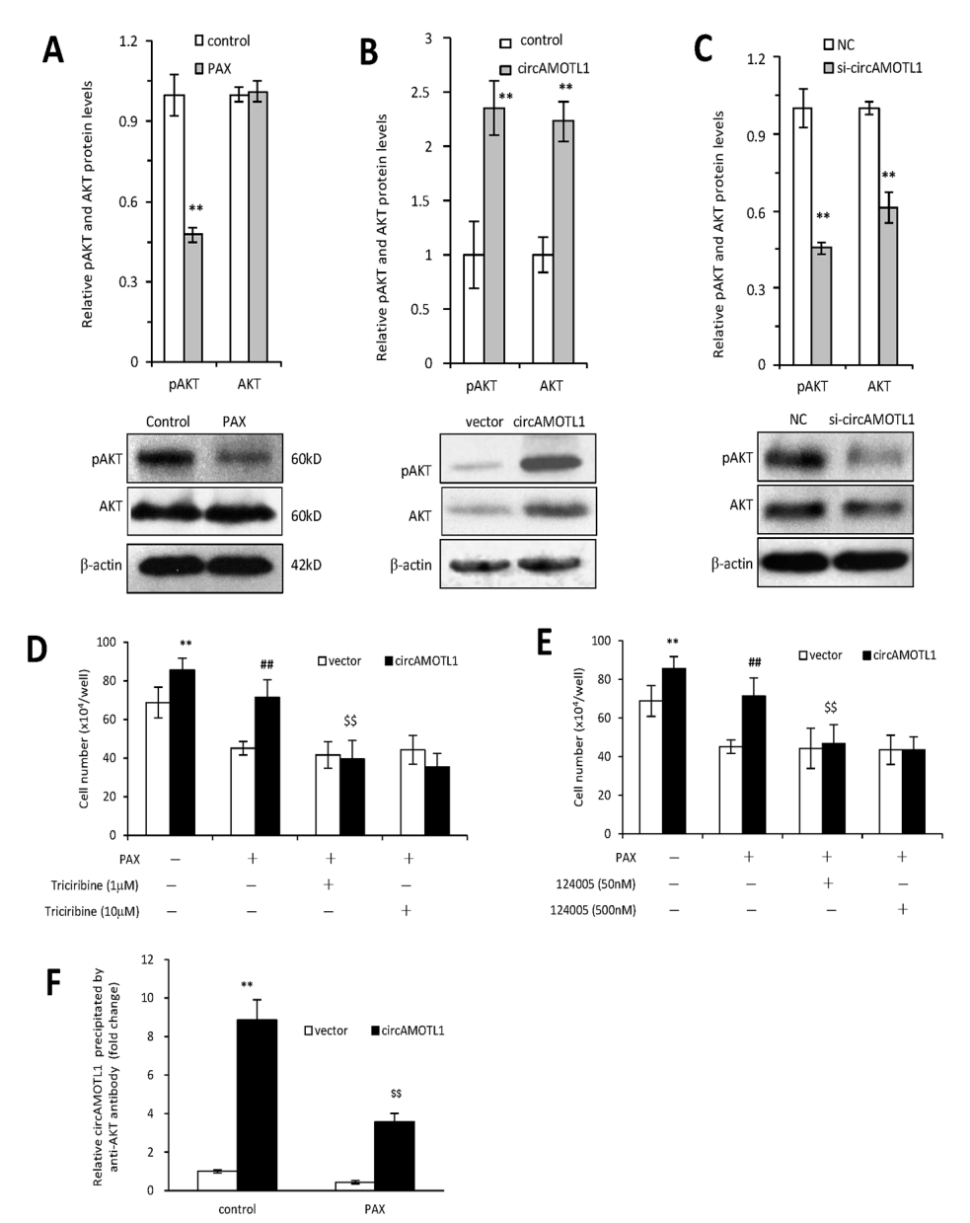
**Role of AKT in circAMOTL1 mediated drug resistance.** (**A**) Upper, the protein expression of AKT and phosphorylated AKT were detected in PAX treated MDA-MB-231 cells and quantified. Lower, typical Western blots are shown. (**B**) Upper, the protein expression of AKT and phosphorylated AKT were detected in vector control and circAMOTL1 transfected cells. Lower, typical Western blots are shown. (**C**) Upper, the protein expression of AKT and phosphorylated AKT were detected in negative control and circAMOTL1 siRNA transfected cells. Lower, typical Western blots are shown. (**D**) AKT inhibitor Triciribine was used at the concentrations of 1 and 10 μM to block the effect of AKT pathway, and cell viability was measured. n=8. **p<0.01 compared to untreated vector control. ^##^p<0.01 compared to PAX-treated vector control. ^$$^p<0.01 compared to untreated circAMOTL1 overexpression group. (**E**) AKT inhibitor, 124005, was used at the concentrations of 50 and 500 nM to block the effect of AKT pathway, and cell viability was measured. n=8. **p<0.01 compared to untreated vector control. ^##^p<0.01 compared to PAX-treated vector control. ^$$^p<0.01 compared to untreated circAMOTL1 overexpression group. (**F**) The circAMOTL1 expression after immunoprecipitation with phosphorylated AKT antibody (Ser473) in PAX treated MDA-MB-231 cells. n=4 **p<0.01 compared to untreated vector control. ^$$^p<0.01 compared to untreated circAMOTL1 overexpression group.

AKT has been well acknowledged as one of the essential mediators for chemoresistance against PAX in cancer cells [25, 26]. Therefore, in the present study, we further examined whether circAMOTL1 mediated the PAX-resistance in breast cancer cells via regulating AKT. First, we explored whether AKT was activated by PAX treatment in the MDA-MB-231 cells by investigating the phosphorylation status of AKT. Our results showed that the protein expression of phosphorylated AKT was inhibited upon PAX treatment (Figure 5A), which suggested that AKT pathway played an important role in PAX-resistant in the MDA-MB-231 cells. Secondly, we asked whether overexpression of circAMOTL1 could affect AKT activity in these breast cancer cells. As shown in Figure 5B, overexpression of circAMOTL1 significantly elevated the protein expression of phosphorylated and total AKT. Meanwhile, blocking circAMOTL1 by siRNA could decrease the phosphorylated and total AKT protein levels (Figure 5C). Thirdly, to further confirm the possible role of AKT in the PAX-resistance, we added two of the AKT pathway inhibitors, Triciribine and 124005, to the PAX-treated cells and found inhibiting AKT activity significantly blocked the effect of circAMOTL1 on cell viability upon PAX treatment (Figure 5D and 5E). To decipher the possible mechanism by which circAMOTL1 posttranscriptionally regulate AKT, we tested whether circAMOTL1 could directly bind with phosphorylated AKT in breast cancer cells. We conducted RNA immunoprecipitation with phosphorylated AKT (Ser473) antibody followed by qPCR to detect circAMOTL1 expression. We found that the binding between circAMOTL1 and phosphorylated AKT was significantly increased in MDA-MB-231 cells with circAMOTL1 overexpression, while such binding could be suppressed by PAX treatment (Figure 5F).

**Figure 6:**
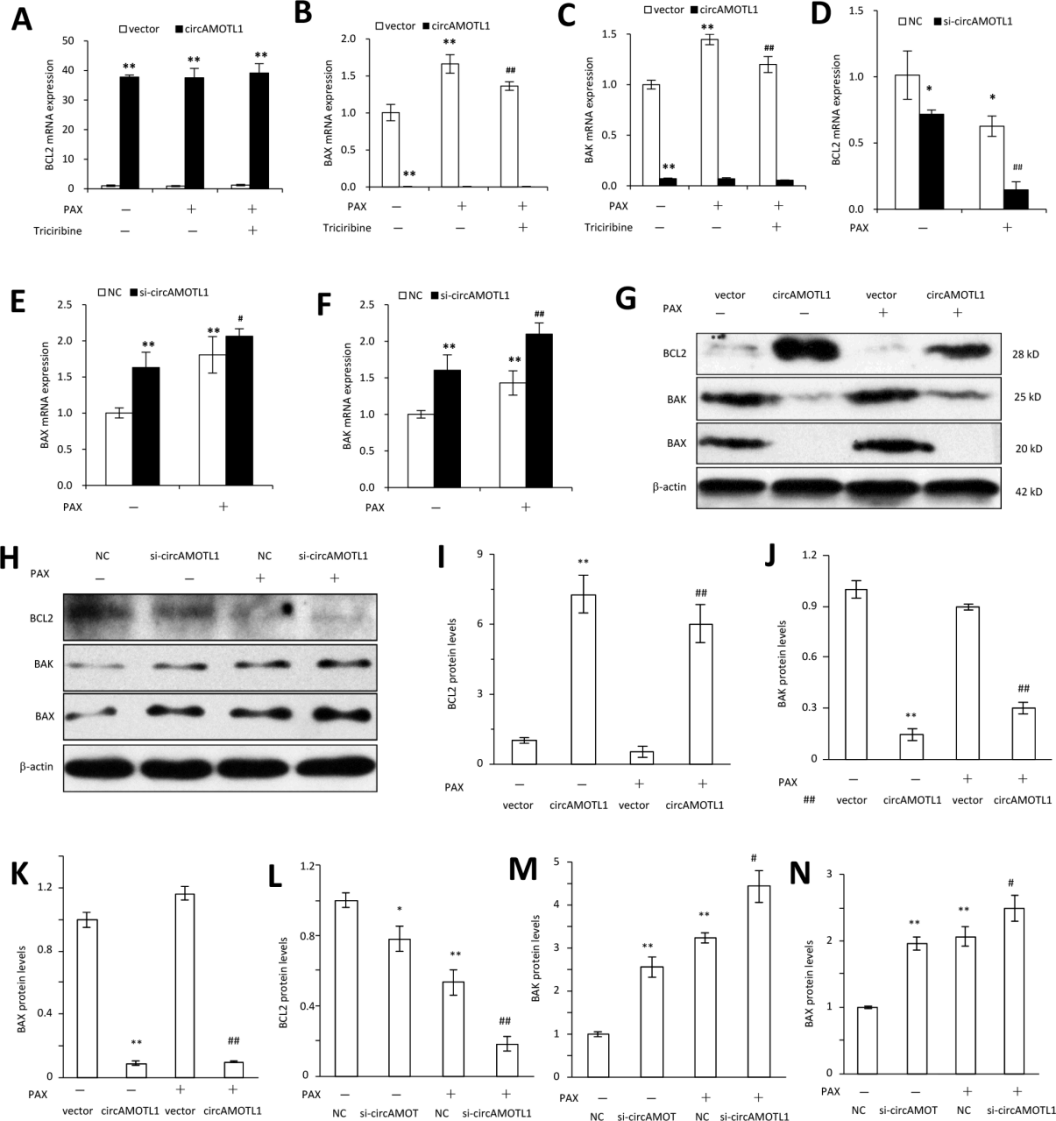
**Effect of circAMOTL1 on apoptosis-associated genes.** MDA-MB-231 cells transfected with or without circAMOTL1 plasmid were treated with PAX (1 μg/ml) for 24 hours. One group of PAX-treated cells were subjected to Triciribine (1 μM) treatment. The mRNA expression of BCL2 (**A**), BAX (**B**) and BAK (**C**) was detected with RT-PCR. n=4. **p<0.01 compared to untreated vector control. ^##^p<0.01 compared to PAX-treated vector control. In addition, MDA-MB-231 cells were also transfected with negative control or circAMOTL1 siRNA (si-circAMOTL1) followed by PAX treatment for 24 hours. The mRNA expression of BCL2 (**D**), BAX (**E**) and BAK (**F**) was detected with RT-PCR. n=4. *p<0.05, **p<0.01 compared to untreated negative control. ^#^p<0.05, ^##^p<0.01 compared to untreated si-circAMOTL1. (**G**) The protein expression of apoptosis-associated proteins, such as BCL2, BAX and BAK was detected in vector control and circAMOTL1-transfected cells treated with or without PAX. (**H**) The protein expression of apoptosis-associated proteins BCL2, BAX and BAK was detected in negative control and circAMOTL1 siRNA-transfected cells treated with or without PAX. (**I**–**K**) Quantitation of the intensities of protein bands of BCL2 (I), BAK (**J**) and BAX (**K**) in Panel G. (**L**–**N**) Quantitation of the intensities of protein bands of BCL2 (**L**), BAK (**M**) and BAX (**N**) in Panel H.

Last, we further explored the mechanisms how circAMOTL1 mediated the chemoresistance in breast cancer cells against PAX by investigating the AKT-related signaling pathway. We examined the gene and protein expression of AKT related pro- and anti-apoptotic genes, including BCL2, BAX and BAK. Our results showed that the gene expression of BAX and BAK was dramatically decreased while BCL2 expression was remarkably boosted in circAMOTL1-overexpressed cells compared to cells transfected with vector control. Moreover, PAX could significantly elevate the expression of BAX and BAK, while such elevation could be partly reversed by AKT inhibitor. In accordance, BCL2 expression was suppressed by PAX in vector control cells while PAX did not affect BCL2 expression in circAMOTL1-overexpressed cells (Figure 6A–6C). To further confirm the effect of circAMOTL1 on the expression of apoptosis-related genes, we measured gene expression of BCL2, BAX and BAK after blocking the circAMOTL1 by siRNA. The expression of BAX and BAK was significantly elevated but BCL2 was decreased upon the inhibition of circAMOTL1 expression by siRNA. After PAX treatment, suppression of circAMOTL1 could increase the BAX and BAK expression but inhibit the BCL2 expression (Figure 6D–6F). Accordingly, the same trends were observed in the protein expression of BCL2, BAX and BAK as the gene expression of them did (Figure 6G and 6H). The protein levels were quantified (Figure 6I–6N). These results suggested that circAMOTL1 down-regulated pro-apoptotic genes and up-regulated anti-apoptotic genes leading to the drug resistance to PAX.

## DISCUSSION

Chemoresistance continues to be the major problem for cancer therapy. In this study, we identified a novel mechanism whereby circAMOTL1 enhanced the resistance of PAX treatment in triple negative breast cancer cells. Our results demonstrated that circAMOTL1 suppressed cell apoptosis and promoted cell survival via activating AKT to produce phosphorylated AKT followed by regulating the pro- and anti-apoptotic gene expression in breast cancer cells.

MDA-MB-231 cell line is a triple negative breast cancer cell model for studying drug resistance in advance breast cancer. It was reported that PAX resistance was easier to develop in MDA-MB-231 cells [28]. In the present study, we first treated MDA-MB-231 cells with three different types of chemotherapy agents which included epirubicin from anthracycline family, cyclophosphamide that is an oxazophorine alkylating agent, and PAX from taxanes family. Among these three agents, PAX induced much less cell death when compared to the other two compounds. By treating the cancer and non-cancer cells with the same dose of PAX, we found that MDA-MB-231 cells were less vulnerable to PAX than non-cancer cell line BEAS2B. According to our previous findings, circAMOTL1 expression was significantly higher in breast cancer tissues compared to those in adjacent benign tissue [12]. Therefore, the expression of circAMOTL1 was examined in several different cell lines to validate whether the sensitivity of cells to PAX was associated with the circAMOTL1 levels. A significantly higher level of circAMOTL1 was observed in two breast cancer cell lines, MDA-MB-231 and MCF-7, and two liver cancer cell lines, HepG2 and SNU449, while the circAMOTL1 expression was relatively much lower in two immortalized non-cancer cell lines, BEAS2B and HaCaT. These results implied that high level of circAMOTL1 might be associated with the low sensitivity of cancer cells to PAX treatment.

Based on the above findings, we postulated that circAMOTL1 might be a key player responsible for the resistance of cancer cells to PAX. As anticipated, after successful transfection of ectopic circAMOTL1 construct into MDA-MB-231 cells, these cells developed stronger PAX resistance compared to cells with vector control in our study. However, no difference was seen on the responses to epirubicin and cyclophosphamide treatment.

To decipher how circAMOTL1 led to the resistance of breast cancer cells against PAX, series of malignant cell behaviors in circAMOTL1-overexpressing cells were profiled. We found circAMOTL1 significantly promoted cell survival, prevented cell apoptosis, and facilitated cell invasion in breast cancer cells. Such above effects were further confirmed by blocking the endogenous expression of circAMOTL1 with its specific siRNA.

After claiming the role of circAMOTL1 in PAX-resistance of breast cancer cells, we also explored the possible underlying mechanisms. Our recent study has demonstrated that circAMOTL1 could bind with AKT and phosphorylated AKT protein and thus increased the cell proliferation and survival of fibroblasts as well as endothelial cells [29]. AKT serves as a checkpoint protein in tumorigenesis by regulating apoptosis, cell cycle progression, angiogenesis and mRNA translation etc [30]. Moreover, AKT has been demonstrated to be one of the most essential mediators that are regulated by PAX [25, 26]. Therefore, the protein expressions of AKT and phosphorylated AKT were measured in the present study. In MDA-MB-231 cells, our results showed that PAX significantly inhibited the phosphorylated AKT expression. These results were in accordance with other studies which proved that phosphorylated AKT protein expression decreased in a time- and dose-dependent manner upon PAX treatment and such inactivation of AKT was required for sensitization to PAX [31]. Then, we tested whether circAMOTL1 could regulate AKT activity by measuring the AKT phosphorylation in MDA-MB-231 cells. We found the total and phosphorylated AKT levels were remarkably elevated in MDA-MB-231 cells transfected with circAMOTL1 plasmids compared to those with vectors. These results validated our hypothesis that circAMOTL1 overexpression induced PAX resistance in cancer cells by regulating the AKT activities. These results also implied that circAMOTL1 may be a key player for the development of PAX resistance in breast cancer cells. We also employed AKT inhibitors along with PAX treatment in circAMOTL1 overexpressing cells, which reduced the cell viability to the comparable levels as those in cells with vector control. These results further confirmed AKT pathway mediated the PAX-resistance in the circAMOTL1-overexpressed cancer cells.

Last but not least, we examined several pro- and anti-apoptotic genes, including BCL2, BAX and BAK, to verify the mechanisms that circAMOTL1 inhibited cell apoptosis. CircAMOTL1 overexpression in cancer cells remarkably increased anti-apoptotic gene BCL2 expression and inhibited pro-apoptotic gene BAX and BAK expression. Although PAX significantly increased the levels of pro-apoptotic genes and decreased the levels of anti-apoptotic genes and Triciribine, the AKT inhibitor, could reverse the effects of PAX in vector control cells, PAX and Triciribine could not further alter the extremely low expression of BAX and BAK and extremely high expression of BCL2 in circAMOTL1 overexpressing cells (Figure 6). Therefore, the strong effects of circAMTOL1 on these pro- and anti-apoptosis genes should be responsible for the PAX resistance in breast cancer cells. In accordance, the changes of protein expression of these apoptosis-associated genes further confirmed our claims (Figure 6).

**Figure 7:**
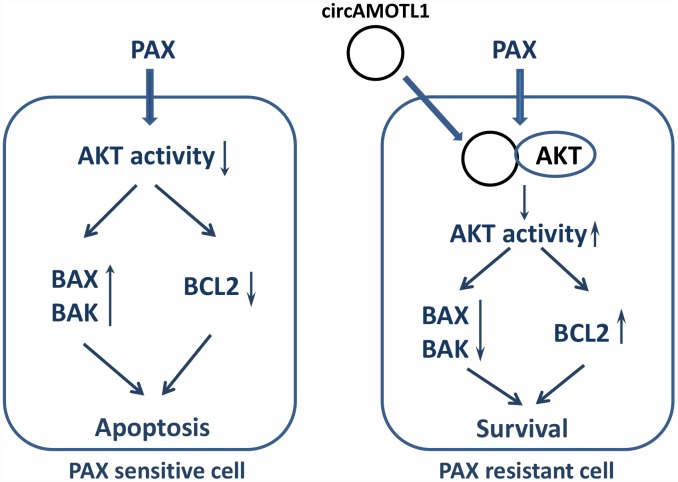
**Proposed mechanism of PAX resistance via circAMOTL1.**

In conclusion, the present study here identified a possible novel target of breast cancer treatment, circAMOTL1. CircAMOTL1 mediated the chemoresistance against PAX in breast cancer cells via posttranscriptional regulation of AKT and led to the changes of AKT related apoptotic genes (Figure 7). Our findings may lead to a better therapeutic approach to improve the sensitivity of breast cancer cells to PAX and reduce the resistance against PAX in its clinical application.

## MATERIALS AND METHODS

### Cell culture

The human breast cancer cells, MDA-MB-231 (American Type Culture Collection) were cultured in Dulbecco's modified Eagle's medium (DMEM) supplemented with 10% fetal bovine serum (FBS). PAX, epirubicin and cyclophosphamide were acquired from the Pharmacy Department of Sunnybrook Hospital. The cells were treated with different concentrations for 24 hours. The AKT inhibitors, Triciribine and 124005, were purchased from EMD Millipore (Burlington, MA).

### Generation of circAMOTL1

The circAMOTL1 construct was synthesized from a basic sequence (flanked by two restriction enzyme sites XhoI and AgeI) containing the 3’-half-intron-enox-II fragment (splice acceptor or SA) of bacteriophage T4 td gene, and a small space sequence, and an exon-I splice donor (SD)-5’-half-intron segment. The basic sequence containing an internal ribosome entry site (IRES) was cloned into the multiple cloning sites of pEGFP-N1, which facilitated the replacement of small space sequence with the circAMOTL1 inserted to generate the circAMOTL1 and GFP. Two restriction sites, HindIII and SalI, in the small space sequence were recognized for digestion and insertion of the circAMOTL1. The vector control plasmid was inserted with a non-related sequence instead of the human circAMOTL1 sequence.

All primers and the anti-circAMOTL1 siRNAs were obtained from Integrated DNA Technologies, Inc. (Coralville, IA, USA). The sequences were listed in Table 1.

**Table 1:** Sequence of primers.

**Primers**	**Forward**	**Reverse**
circAMOTL1	5′-cagcctgtgagaacagatgtggcc-3′	5′-ggttggggtgccataccgcagttg-3′
BCL2	5′-gtcgcagaggggctacgagtggga-3′	5′-accacaggtggcaccgggctgagc-3′
BAX	5′-gtgcctcaggatgcgtccaccaag-3′	5′-ggcaaagtagaaaagggcgacaac-3′
BAK	5′-gagtgtggagagcctgccctgccc-3′	5′-ctgcaggtgctgcaacatggtctg-3′

### Real-time PCR

The expression of circAMOTL1 and the mRNA expression of BCL2, BAK and BAX were determined by reverse transcription and real-time PCR analysis using CFX connect system (Bio-Rad) and normalized with housekeeping gene. In brief, total RNA was extracted from cells using GENEzol TriRNA pure kit. Total RNA (1 μg) was converted to cDNA by reverse transcription. The real-time PCR reaction mixture contained 0.4 μM of forward and reverse primers and 1 μl of cDNA product in SYBR green supermix reagent (Bio-Rad).

### Cell viability assay

Cell viability assay was conducted as previously described [32]. Cells (1× 10^5^) were seeded onto 6-well dishes in DMEM medium containing 10% FBS and maintained at 37°C overnight. For measuring the cell viability, cells were treated with different concentrations of drugs and then the cell number was determined with a coulter counter. The half maximal inhibitory concentration (IC50) of PAX in MDA-MB-231 cells was determined by using cell counting kit-8 (Cedarlane) as previously described [33].

### Cell invasion assay

Cells (1×10^5^ per well) were suspended in serum-free medium and loaded into transwell membrane inserts (Corning) that were pre-coated with 50 μl of 10% Matrigel. The inserts were placed in 24-well plates containing medium supplemented with 10% FBS. Cells were incubated at 37 °C and allowed to invade through the Matrigel and the membrane pores in the inserts. The upper Matrigel layer and cells were removed 48 h after cell inoculation. The cells that invaded to the lower side of the membrane were fixed with methanol and stained with Coomassie brilliant blue. Cells on the lower surface were counted from representative areas for quantification.

### Annexin V/ PI staining and FACS analysis

To determine the cell apoptosis, annexin V/PI staining were conducted using eBioscience Annexin V Apoptosis Detection Kit APC (Invitrogen). In brief, cells were washed in PBS and then re-suspended in 1x binding buffer followed by incubation with 1/20 volume of fluorochrome-conjugated Annexin V at room temperature for 10-15 min. Then, the cells were washed with 1x binding buffer and stained with in propidium iodide (PI) followed by flow cytometry analysis as previously described [20].

### Western blot

The protein levels of AKT, phosphorylated AKT, BCL2, BAK and BAX were determined by Western immunoblotting analysis as previously described [34]. In brief, proteins isolated from cells (50 μg) were loaded and separated by electrophoresis on a 10% SDS polyacrylamide gel and then transferred to a nitrocellulose membrane. The membrane was probed with either rabbit anti-Akt antibody (Cell Signaling Technology), rabbit anti-phosphorylated AKT antibody (Cell Signaling Technology), rabbit anti-BAK antibody (Cell Signaling Technology), rabbit anti-BAX antibody (Cell Signaling Technology), or mouse anti-BCL2 antibody (Neomarkers). All antibodies were diluted at 1:1000. HRP-conjugated anti-rabbit or anti-mouse IgG antibody (Jackson ImmunoResearch) was used as the secondary antibody. The corresponding protein bands were visualized using enhanced chemiluminescence reagents. The same membranes were re-probed with rabbit anti-β-actin monoclonal antibody (Abcam) to confirm equal loading of proteins for each sample.

### RNA immunoprecipitation

RNA immunoprecipitation was used to determine the binding of RNA and protein as described [35]. Briefly, cells were lysed in 200 μl co-IP buffer. The Magnetic beads (Surebeads, BioRad) were washed with PBST (PBS containing 1% Tween 20) and incubated with 5 μg of anti-phosphorylated AKT (Ser 473) antibody (Cell Signaling) at room temperature for 10 min. After washing, the beads were mixed with protein lysis and incubated for another 1 hour. Then the beads were washed 3 times with PBST and re-suspended in 0.5 ml TriZol (Invitrogen). The eluted co-precipitated RNA in the aqueous solution was subject to RT-qPCR analysis to demonstrate the presence of the binding products using circAMOTL1 primers.

### Statistical analysis

Results were analyzed by a two-tailed Student's t-test or one-way analysis of variance (ANOVA) followed by Newman-Keuls post-test using Prism 6 (GraphPad Software, Inc., La Jolla, CA). The levels of significance were set at *P < 0.05 and **P < 0.01.
