# Correction for: Posttranscriptional regulation of AKT by circular RNA angiomotin-like 1 mediates chemoresistance against paclitaxel in breast cancer cells

**DOI:** 10.18632/aging.206315

**Published:** 2025-08-31

**Authors:** Jian Ma, Ling Fang, Qi Yang, Steven Hibberd, William W. Du, Nan Wu, Burton B. Yang

**Affiliations:** 1 Sunnybrook Research Institute, Sunnybrook Health Sciences Centre, Toronto, Canada; 2 Department of Urology, The Affiliated Yantai Yuhuangding Hospital of Qingdao University, Yantai, China; 3 The First Hospital, Jilin University, Jilin, China; 4 Department of Laboratory Medicine and Pathobiology, University of Toronto, Toronto, Canada

**Keywords:** circular RNA, circAMOTL1, chemoresistance, PAX, AKT

**This article has been corrected:** The authors have requested a replacement for Figure 4D. A representative image of the si-circAMOTL1 Control sample in Figure 4D was mistakenly mixed up. The correct panel has now been prepared with the si-circAMOTL1 Control image from the original set of experiments. This alteration does not affect the results or conclusions of this work.

The correct Figures 4D is presented below:

**Figure 4:**
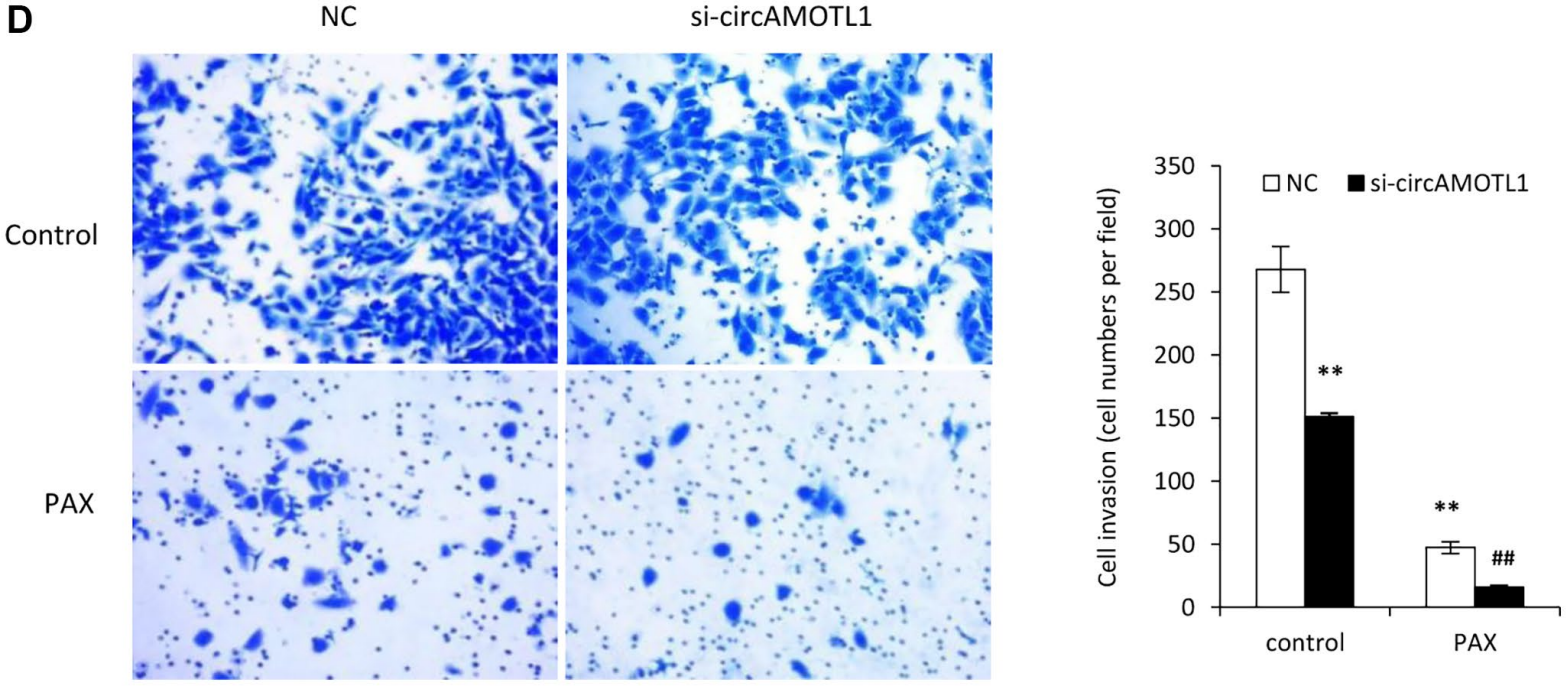
**Effect of circAMOTL1 siRNA on PAX treatment.** MDA-MB-231 cells transfected with negative control or circAMOTL1 siRNA (si-circAMOTL1) were treated with 1 μg/ml PAX for 24 hours. … ((**D**) Cell invasive ability upon PAX treatment was measured with Matrigel invasion assay. Experiments were performed in triplicate. ^**^p < 0.01 compared to untreated negative control. ^##^*p* < 0.01 compared to untreated si-circAMOTL1.

